# Covalent Patterning of Graphene for Controllable Functionalization from Microscale to Nanoscale: A Mini-Review

**DOI:** 10.3389/fchem.2022.829614

**Published:** 2022-03-11

**Authors:** Zhi Li, Kai Li, Shuang Wang, Chao Teng

**Affiliations:** ^1^ Institute of Marine Biomedicine, Shenzhen Polytechnic, Shenzhen, China; ^2^ National Laboratory of Solid State Microstructures, College of Engineering and Applied Sciences, Nanjing University, Nanjing, China

**Keywords:** covalent patterning, electrochemical writing, laser writing, template-directed growth, tip-induced nanoshaving

## Abstract

Covalent patterning of graphene opens many application possibilities in the field of photonics, electronics, sensors, and catalysis due to order-dependent optical properties, band structure engineering, and processibility and reactivity improvement. Owing to the low reactivity of the graphene basal plane, harsh reagents (e.g., radicals) used for covalent functionalization normally result in poor spatial control, which largely compromises the intrinsic properties of graphene. Therefore, precisely spatial control on covalent patterning of graphene is of great importance. Herein, we summarize recent advances for covalent patterning of graphene from the microscale to nanoscale resolution using different techniques such as laser or electrochemical writing, template-directed growth, and tip-induced nanoshaving.

## Introduction

Graphene is a two-dimensional (2D) nanomaterial with sp^2^ hybridized carbon atoms arranged in a honeycomb structure. Since its first mechanical exfoliation as a freestanding single-layer sheet from graphite by Novoselov and Geim in 2004 (Novoselov et al., 2004), graphene has been attracting tremendous scientific attention because of its outstanding mechanical, optical, thermal, and electronic properties, which surpass most of the existing materials. Most importantly, graphene can be considered as a zero-band-gap semiconductor or semi-metal, and its conduction and valence bands intersect at six Dirac points, around which the energy dispersion is linear (Novoselov et al., 2005; Zhang et al., 2005). The unique band structure, on one hand, grants high-speed data processing ability to graphene-based transistors. However, the absence of the band gap, on the other hand, is a large obstacle to turn off the current flow of graphene transistors. Furthermore, the poor processibility and the basal plane reactivity of graphene are also the main problems for potential applications.

One of many effective ways to solve the aforementioned issues is the chemical functionalization of graphene. Graphene surface modification can be achieved *via* both non-covalent and covalent methods. The non-covalent method of graphene functionalization has limited stability. The physisorbed networks, held together through weak non-covalent interactions, hinder its application in graphene-based electronics. In contrast, covalent functionalization is rather robust by forming stable covalent bonds between functional moieties and graphene surfaces. Covalent functionalization of graphene has been proven to be able to open up its band gap (Zhang et al., 2011; Shih et al., 2013), enhance its chemical reactivity (Bissett et al., 2013), and improve its processibility (Englert et al., 2011). However, highly reactive reagents (e.g., radicals) must be used for covalent modification of the inert basal plane of graphene, generally leading to poor spatial control (Kariuki and McDermott, 1999; Greenwood et al., 2015). Fan *et al.* demonstrated that covalent modification of graphene with 4-nitrobenzenediazonium salt led to a significant degradation of its electron transfer characteristics such as reduction in the minimum conductivity, breakdown of electron-hole mobility symmetry, and decrease in the electron-hole mobility (Fan et al., 2010). Current challenges accordingly lie in simultaneously functionalizing graphene and maintaining its excellent properties. Through the density functional theory (DFT), parameterized tight-binding model, and real-space Kubo-Greenwood formalism, it was theoretically predicted that covalent nanopatterning of graphene effectively created nanostripped structures with a tunable band gap, and the electron transport remained unaffected and confined in the so-created quasi-one-dimensional semiconducting channels (Lian et al., 2016). Therefore, efficiently controlling the long-range orderness of covalent functionalization of graphene is of major importance for fundamental research studies and potential applications.

In this mini-review, we discuss the recent progress achieved in the field of spatially controlled covalent nanopatterning of graphitic substrates *via* radical chemistry. We focus on the state-of-art diverse fashions on covalent patterning of graphitic surfaces from micrometer to nanometer precision.

## Covalent Patterning With Microscale Periodicity

**FIGURE 1:**
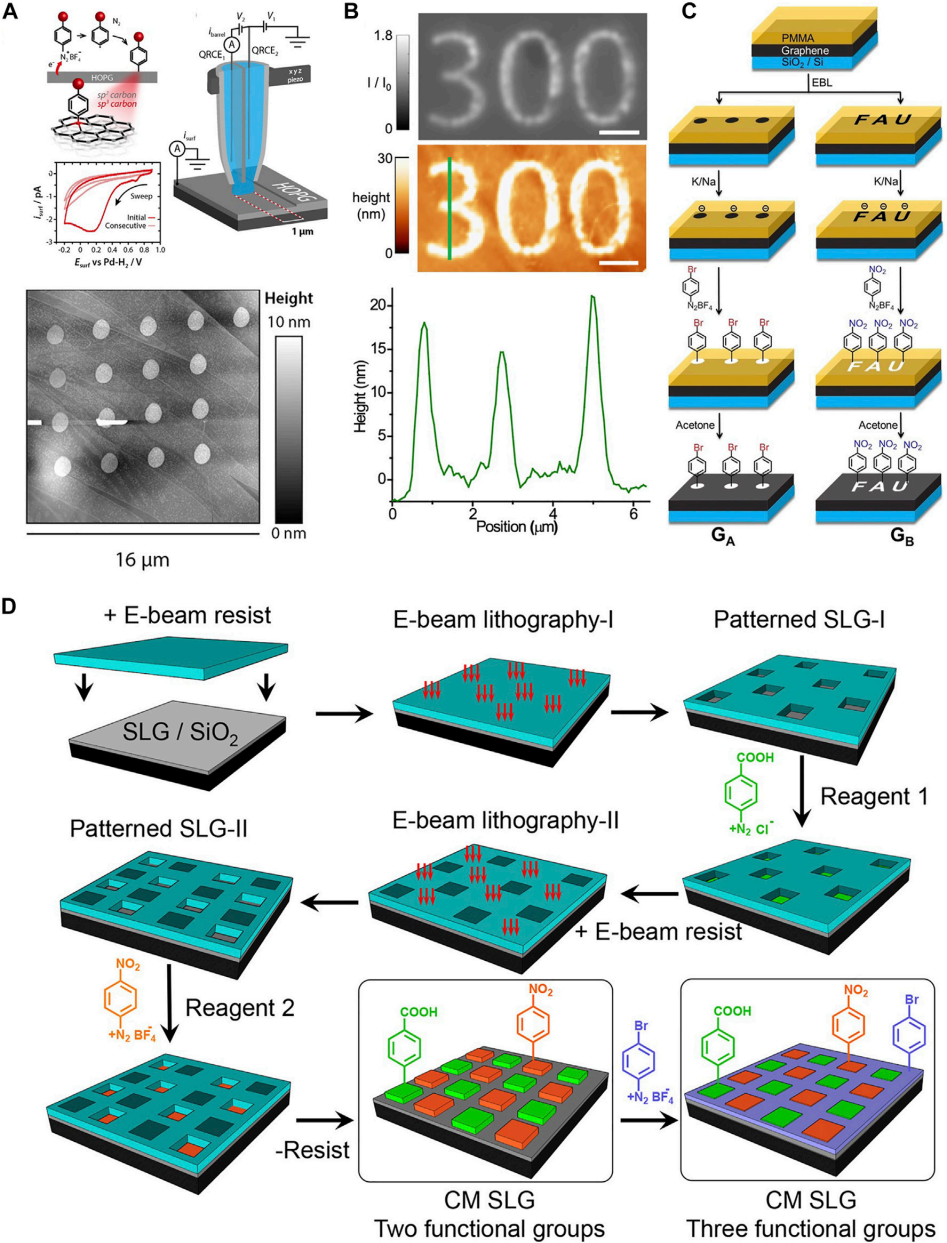
Covalent patterning of graphene with a microscale patterning resolution. **(A)** Schematic representation of the local electrochemical reduction of diazonium modification at an HOPG electrode surface using a scanning electrochemical cell microscopy (SECCM) setup, resulting in the an sp^3^ carbon in the HOPG surface layer and the corresponding AFM image of covalent patterning on the HOPG surface. Panel adapted with permission from Kirkman et al., (2013). Copyright: American Chemical Society. **(B)** The interference reflection microscopy, AFM images, and the corresponding line profile of template-assisted laser writing for covalent patterning of graphene, achieving ∼400 nm patterning resolution. Scale bar = 2 µm. Panel adapted with permission from Li et al., (2019). Copyright: American Chemical Society. **(C)** Schematic illustration of the reaction mechanism for the template-assisted covalent patterning monolayer graphene *via* electron-beam lithography. The diameter of a dot is 5 μm, and the length and width of the FAU logo are 20 and 30 μm, respectively. Panel adapted with permission from Wei et al., (2020). Copyright: John Wiley and Sons. **(D)** Schematic illustration for the sequential covalent patterning of graphene with multiple diazonium components using the combined technique of electron beam lithography and self-limiting diazonium chemistry. Panel adapted with permission from González et al., (2021). Copyright: American Chemical Society.

Some decent efforts have been devoted to the scalable patterning of graphene substrates *via* covalent chemistry, including electrochemical or laser “writing” and locally and globally template-assisted lithography. For example, Kirkman et al., (2013) achieved covalent patterning of a highly oriented pyrolytic graphite (HOPG) surface *via* periodically moving a scanning electrochemical cell microscopy (SECCM) setup after conducting local functionalization with *in situ* generated radicals from electrochemical reduction of diazonium salt **(** Figure 1A). The diameter of each dot and its thickness can be controlled by the size of the cell and the duration of electrochemical activation, respectively. The resulting resolution of covalent patterning was at a micrometer scale. Laser activation is also another efficient method for covalent patterning of graphene. Li et al., (2019) demonstrated a direct covalent patterning method *via* light-induced reduction of diazonium salts on graphene with the assistance of a photoresist template. A photoresist-masked graphene sample was first immersed in an aqueous solution of 4-nitrobenzenediazonium tetrafluoroborate, followed by exposure to blue light for 120 s. Figure 1B shows the interference reflection microscopy and AFM images of covalent patterning of graphene with pattern “300.” The AFM line profile disclosed that the patterning resolution was about 400 nm based on the full width at a half-maximum of three peaks (371, 419, and 408 nm). Wei et al., (2020) proposed another efficient laser-writing protocol for reducing diazonium salts on graphene to conduct covalent patterning of graphene at the micrometer scale (Figure 1C). In this research, the SiO_2_/Si-supported graphene surface was initially covered by a thin polymethylmethacrylate (PMMA) template with desired patterns, followed by the reductive activation of the regions of unprotected graphene with a Na/K alloy electron beam. Afterward, the sample was exposed to the nitro- or bromo-benzenediazonium salt dissolved in ethanol for covalent functionalization. After removing the excess reagent and the PMMA template, covalent patterning of graphene was successfully achieved with a resolution of few micrometers. By applying thermal treatment of the sample at 400°C, the graphene surface was totally recovered by the complete defunctionalization process, which realized a complete write/store/erase cycle for the management of chemical information. Recently, multifunctional graphene was achieved by stepwise covalent patterning of graphene with multiple diazonium components using a combined technique of electron beam lithography and self-limiting diazonium grafting (González et al., 2021). Figure 1D shows the sequential fabrication process for the covalent patterning of graphene with multiple components using the self-limiting diazonium grafting method. SiO_2_-supported graphene was first covered by a PMMA template *via* the spin-coating process, followed by electron beam lithography to form the first pattern with 5 μm × 5 μm for each corral. Afterward, adding the first diazonium solution onto the ascorbic acid-precovered patterned graphene led to the covalent patterning of graphene with the first component. After removing the physisorbed species and the PMMA residues, covalent patterning of graphene with the second diazonium component started over by creating patterns in the unmodified areas. It is worth mentioning that the alignment markers in the SiO_2_ substrate can avoid the overlap of each pattern. The third diazonium component was added to covalently functionalize the rest of the graphene surface, thus leading to generate covalently patterned graphene with three functional components. Besides using diazonium salts as radical sources, hypervalent iodine compounds have also been applied for covalent functionalization of graphene. Bao et al., (2021) applied a laser-writing process for covalent patterning of the graphene substrate. A drop of hypervalent iodine solution was first deposited on a SiO_2_/Si-supported graphene. Afterward, a green laser light was used to decompose the hypervalent iodine reagents for the generation of highly reactive radicals, which locally modified the graphene surface by forming covalent bonds. Desired patterns with micrometer resolution can be achieved by a step moving of the laser source. Cleavage of dibenzoylperoxide (DBPO) *via* thermal or photoactivation was also an efficient way to produce radicals for graphene covalent functionalization. Gao et al., (2016) demonstrated an effective method of covalent functionalization of graphene *via* heat-initiated cleavage of DBPO with the assistance of a polydimethylsiloxane (PDMS) template. The DBPO decomposed at around 80°C to aryl radicals, which subsequently attached the exposed surface of graphene, forming covalent patterns at the micrometer scale. Four years later, Edelthalhammer et al., (2020) modified the method by locally cleaving the DBPO compounds on a graphene substrate *via* laser-writing activation, achieving a pattering resolution of 2 μm. Surprisingly, the covalent functionalization process was totally reversible, which allowed the write/read/erase control over the covalent chemical information stored on the graphene surface. In other words, applying a 532 nm laser source on the DBPO precovered graphene enabled writing of chemical information on the substrate. Switching the wavelength of the laser light to 633 nm showed better Raman imaging resolution, providing an excellent opportunity of reading the chemical information of the 2D-patterned graphene. The erasing process was simply performed by thermally removing the covalently attached molecules at high temperatures.

## Covalent Patterning With Nanoscale Periodicity

**FIGURE 2:**
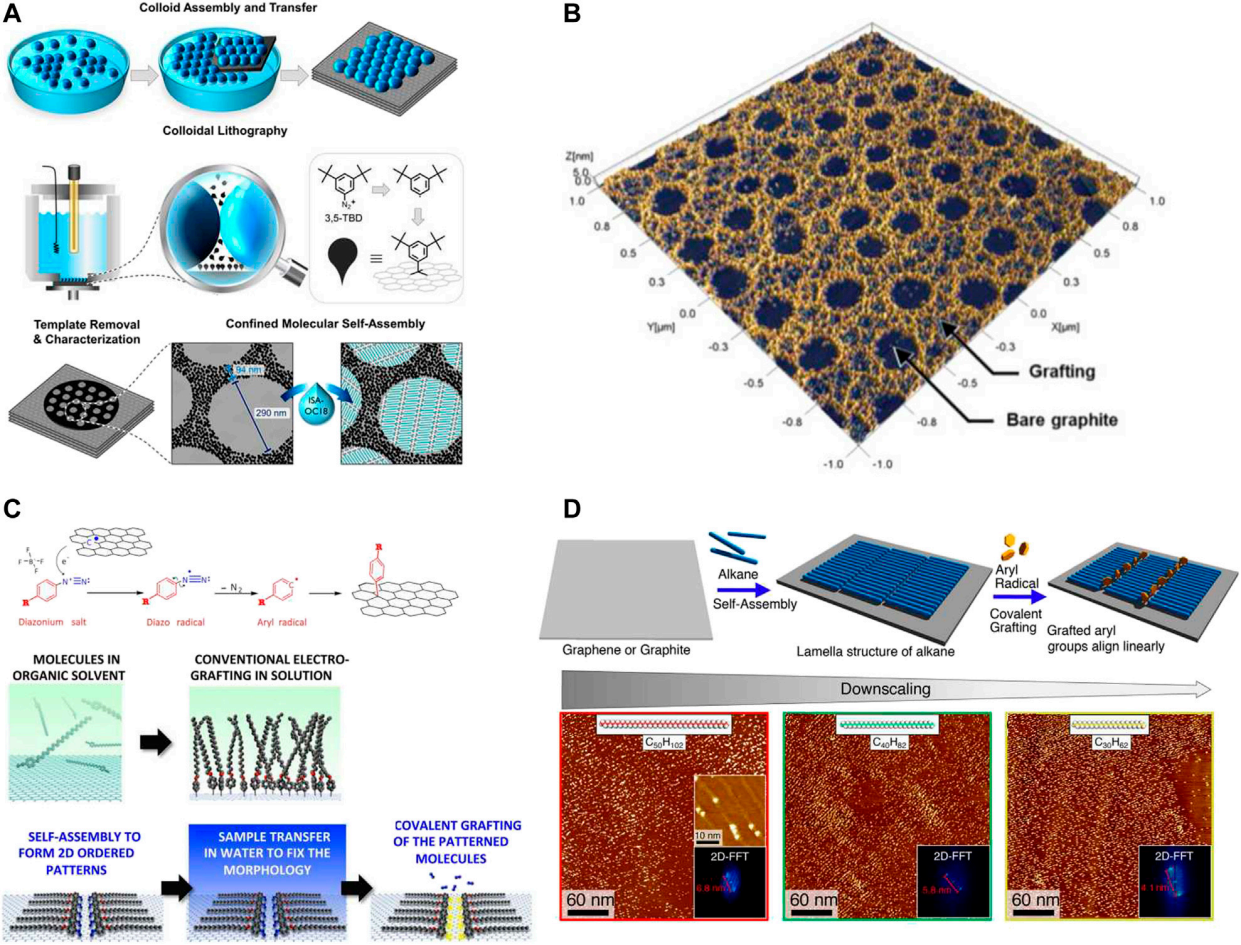
Covalent patterning of graphene with a nanoscale patterning resolution. **(A)** Schematic demonstration of the colloidal lithography method to generate covalently formed nanocorrals on the HOPG substrate for confined molecular self-assembly. Panel adapted with permission from Van Gorp et al., (2018). Copyright: American Chemical Society. **(B)** Covalent patterning of graphitic substrates with the assistance of electrochemically *in situ*-generated products and nanobubbles as the template. Panel adapted with permission from Phan et al., (2019). Copyright: American Chemical Society. **(C)** Schematic illustration of covalent patterning on graphitic surfaces *via* the pre-assembled monolayer of p-(n-octadecyloxy) benzene diazonium, followed by electrochemical activation. Panel adapted with permission from Xia et al., (2016). Copyright: American Chemical Society. **(D)** Schematic illustration of covalent patterning of HOPG using self-assembled monolayers of alkanes with tunable length as templates and corresponding STM images with Fourier transforms. Panel adapted with permission from Tahara et al., (2018). Copyright: American Chemical Society.

Patterning graphene at the nanoscale resolution is of critical importance to meet the requirement of the modern electronics. Some brilliant methods were applied to the covalent functionalization of the graphene surface with the resolution at a few nanometers. Van Gorp et al., (2018) applied the colloid assembly method of forming an ordered template with polystyrene beads, which was then transferred onto a graphite surface as the mask for periodic covalent functionalization. The beads floated at the air–liquid interface and were left undisturbed for 4 h to form a template *via* self-assembly (Figure 2A). The assembled beads were then transferred to the HOPG substrate by a careful scooping method to achieve a monolayer template. After drying with Ar, the bead-masked HOPG was then covalently functionalized in a three-electrode electrochemical cell *via* electrochemical reduction of diazonium. After removing the physisorbed molecules and the beads, covalently functionalized nanocorrals with 290 nm in diameter were fabricated on the HOPG surface. Surprisingly, a composite of *in situ*-generated side-products and nanobubbles is also able to achieve covalent patterning of graphitic substrates with nanoscale periodicity over large areas. Phan et al., (2019) demonstrated a convenient covalent functionalization approach for nanopatterning graphite and graphene substrates by electrochemically activated dediazotization of a mixture of two aryl diazonium compounds (Figure 2B). The mechanism was based on the electrochemical formation of side-product-stabilized nanobubbles that worked as templates for covalent nanopatterning of graphitic substrates. The average diameter of the nanobubbles can be controlled in the range between 45 and 130 nm *via* adjusting the overall concentration, the ratio of two aryl diazonium compounds, or the applied potential. Sub-10 nm patterning resolution was also realized with the assistance of the molecular self-assembly process. Xia et al., (2016) presented a versatile two-step approach for covalent patterning of graphene at the nanometer scale (Figure 2C). An ordered molecular monolayer of 4-docosyloxy-benzenediazonium was first formed *via* a self-assembly process at the solid–liquid interface on the graphene surface. After removing the organic solvent, the ordered 4-docosyloxy-benzenediazonium monolayer was immobilized on the graphene surface. Applying an electrochemically reductive impulse activated the diazonium groups to radicals, which subsequently attached the graphene surface to form covalent bonds. The resulting periodicity of the covalent functionalization sites remained the same to the pre-programmed periodicity determined by the length of the alkoxyl chain of 4-docosyloxy-benzenediazonium. Therefore, it is possible to control the periodicity of the covalent patterning of graphene *via* adjusting the alkoxyl chain length. The template-assisted method is also feasible for covalent nanopatterning of graphitic substrates. After realizing that an ultra-thin single layer of self-assembled n-alkanes can prevent the underneath graphitic substrates from being attacked by radicals, Li et al., (2017) and Tahara et al., (2018) employed self-assembled monolayers of n-alkanes as linear masks for directing covalent functionalization of graphitic substrates with a lateral periodicity of 5 or 7 nm according to the length of the n-alkanes (Figure 2D). Different from the previous approach (Xia et al., 2016) that requires programmed diazonium molecules with long alkoxyl chains, this method is feasible for covalently functionalizing graphitic substrates with all sorts of diazonium molecules. Lee et al., (2011) successfully created a patterned graphene with a 35 nm channel for the fabrication of graphene-based field-effect transistors. A thin film of polystyrene was first deposited on the graphene substrate as a mask by scanning probe lithography, followed by exposing the masked sample to XeF_2_ gas for fluorination. The unmasked part of graphene was converted to insulating fluorographene, while leaving a 35 nm channel of pristine graphene. Importantly, this fluorination strategy did not compromise the carrier mobility of the graphene channel, reaching 2,692 cm^2^/(Vs).

## Tip-Induced Covalent Patterning

Besides the aforementioned bottom-up approach for covalent nanopatterning, the top-down method is also feasible for creating nanopatterns on the pre-grafted graphitic substrates. At the earliest, Xu and Liu, (1997) demonstrated that the grafted thiol molecules were able to be removed from the Au surface by nanoshaving with an AFM tip. When the AFM tip was scanning on the Au surface, molecules were removed to create an empty area depending on the scanning dimensions. Greenwood et al., (2015) demonstrated the feasibility of recovering graphitic substrates by removing covalently grafted molecules from the graphitic surfaces *via* nanoshaving with the tip of the scanning tunneling microscope (STM). A “nano-man” was created by adjusting the nanoshaving angle and the dimensions. Following this method, Verstraete et al., (2016), applied the covalently nanopatterned surface to the study of molecular self-assembly behavior under nano-confinement conditions. The authors found that the slow nanoshaving direction can induce the molecular alignment direction in the *in situ* pattern, while the multiple directions were observed during the molecular self-assembly process in the *ex situ* pattern. This finding provides a possibility of controlling molecular crystallization in the desired alignment direction. The corral size of patterns can be controlled by the STM scanning area ranging from 11 to 67 nm.

## Discussion

Covalent functionalization of graphene has been intensively studied in order to widen the application range and improve the chemical reactivity and processibility of graphene. However, precise spatial control on the covalent patterning of graphene has been a challenge. This work provides the overview of covalent patterning of graphene from the microscale to nanoscale including laser or electrochemical writing, template-directed growth, and tip-induced nanoshaving, which shows a great potential for achieving decent resolution even down to few nanometers. This offers an opportunity toward a wide range of applications. For example, the nano-corrals generated on graphitic substrates can be used as microelectrodes for catalytic uses, such as biosensors, or for molecular recognition. Studying the *in situ* growth process of the molecular self-assembly is possible by STM tip-induced nanoshaving. The covalently patterned arrays can potentially introduce a band gap in graphene by maintaining one-dimensional high-speed electron channels. Moreover, further grafting another type of molecule in the empty spaces in the nanopatterned graphene can lead to heterogeneous functionalities for creating hydrophilicity–hydrophobicity or electron donating–electron withdrawing heterostructures for electronic and optoelectronic applications.

## Fundings

This research was funded by the Shenzhen Science and Technology Innovation Commission (GJHZ20190819151807167 and JSGG20200103094001790) and the Post-doctoral Foundation Project of Shenzhen Polytechnic (grant no. 6020330009K, grant no. 6020330008K, and grant no.6021330004K).
